# NT-proBNP as Early Marker of Subclinical Late Cardiotoxicity after Doxorubicin Therapy and Mediastinal Irradiation in Childhood Cancer Survivors

**DOI:** 10.1155/2015/513219

**Published:** 2015-04-16

**Authors:** Amal Zidan, Laila M. Sherief, Amera El-sheikh, Safaa H. Saleh, Doaa A. Shahbah, Naglaa M. Kamal, Hanan S. Sherbiny, Heba Ahmad

**Affiliations:** ^1^Clinical Pathology Department, Faculty of Medicine, Zagazig University, Zagazig 44519, Egypt; ^2^Pediatric Department, Faculty of Medicine, Zagazig University, Zagazig 44519, Egypt; ^3^Pediatric Department, Faculty of Medicine, Cairo University, Cairo 11562, Egypt

## Abstract

*Background*. Childhood cancer survivors treated with anthracyclines and mediastinal irradiation are at risk for late onset cardiotoxicity. *Aims of the Study*. To assess the role of N-terminal pro-brain natriuretic peptide (NT-proBNP) and tissue Doppler imaging (TDI) as early predictors of late onset cardiotoxicity in asymptomatic survivors of childhood cancer treated with doxorubicin with or without mediastinal irradiation. *Methods*. A cross-sectional study on 58 asymptomatic survivors of childhood cancer who received doxorubicin in their treatment protocols and 32 asymptomatic Hodgkin's lymphoma survivors who received anthracycline and mediastinal irradiation. Levels of NT-proBNP, TDI, and conventional echocardiography were determined. Results. Thirty percent of survivors had abnormal NT-proBNP levels. It was significantly related to age at diagnosis, duration of follow-up, and cumulative dose of doxorubicin. TDI detected myocardial affection in 20% more than conventional echocardiography. Furthermore, abnormalities in TDI and NT-pro-BNP levels were more common in Hodgkin lymphoma survivors receiving both chemotherapy and radiotherapy. *Conclusions*. TDI could detect early cardiac dysfunction even in those with normal conventional echocardiography. Measurement of NT-proBNP represents an interesting strategy for detecting subclinical cardiotoxicity. We recommend prospective and multicenter studies to validate the role of NT-proBNP as an early marker for late onset doxorubicin-induced cardiotoxicity.

## 1. Introduction

Anthracyclines are widely used antineoplastic agents for the treatment of both childhood hematological malignancies and solid tumors, including acute lymphoblastic leukemia (ALL), acute myeloid leukemia, non-Hodgkin and Hodgkin's lymphoma, neuroblastoma, osteosarcoma, Ewing tumors, and nephroblastoma. Almost 60% of children with cancer receive anthracyclines as a part of their treatment [1]. A major limitation of anthracycline is the risk of cardiotoxicity, manifested as asymptomatic cardiac dysfunction in up to 57% [2, 3] and cardiomyopathy with subsequent clinical heart failure in up to 16% [4]. Subclinical cardiac abnormalities are persistent and progressive after anthracycline therapy and can lead to significant clinical symptoms [5]. Early and accurate diagnosis of ventricular dysfunction in asymptomatic cardiac patients may permit a prompt onset of therapy of subclinical cardiotoxicity before the development of life-threatening complication [6].

Current monitoring techniques, such as MUGA (multigated acquisition scan) or echocardiography, have substantial limitations and detect LV dysfunction only after it had occurred. Cardiotoxicity is usually diagnosed only upon manifestation of clinical signs and symptoms or progressive cardiac dysfunction. Thus, new diagnostic tests are required to confirm ventricular dysfunction induced by anticancer therapy [6]. It has been suggested that NT-proBNP may be useful in early detection of myocardial damage after anticancer therapy [7–9]. However, few data have been published demonstrating the usefulness of NT-proBNP in detection of late onset cardiotoxicity occurring several years after completion of chemotherapy in childhood cancer survivors [10].

This study aimed to assess the role of N-terminal pro-brain natriuretic peptide (NT-proBNP) and tissue Doppler imaging (TDI) as early predictors of late onset cardiotoxicity in asymptomatic survivors of childhood cancer treated with doxorubicin with or without mediastinal irradiation.

## 2. Methods

This cross-sectional study was carried out on 80 asymptomatic survivors of childhood cancer, who visited the late effects clinics of Pediatric Oncology, Zagazig University Hospitals, and late effects clinics of Pediatric Oncology, Benha Specialized Pediatric Hospital, during the period from January 2011 to December 2013. All children received doxorubicin as a part of their therapy for various kinds of malignancy for more than one year. Informed consent was obtained from the patients or their parents. Our inclusion criteria were patients in a stable general condition with no signs or symptoms of cardiac impairment at the time of evaluation and normal hepatic and renal function tests.

Patients with history of cardiac diseases and hypertension were excluded from the study. The study was approved by the local ethics committee of contributing hospitals.

All survivors underwent all the following.

1. Complete history taking and thorough physical examination including weight, body surface area (BSA), and measurement of blood pressure.
2. The cumulative dose of anthracycline calculated for each patient according to his BSA.
3. Methods for evaluation of subclinical cardiotoxicity.

*(a) Conventional Echocardiography.* Detailed conventional echocardiography was performed at Pediatric Cardiology Unit, Zagazig University Hospitals, using (Vivid 7 Pro, 7 MHz and 3 MHz transducer, GE, Horten, Norway). Echocardiography was performed by a cardiologist who was blinded to the clinical details. Two-dimensional, M-mode, pulsed-wave (PW), and continuous-wave Doppler echocardiographic images were acquired. For all patients, standard measurements were left ventricular posterior wall thickness at diastole (LVPW), interventricular septum thickness at diastole (IVS), left ventricular dimensions at end-systole (LVES), and left ventricular dimensions at end-diastole (LVED). Shortening fraction (FS) and ejection fraction (EF) of the LV were calculated from M-mode measurements of LV dimensions at the level of mitral valve leaflets in parasternal long-axis view. Sample volume of the PW Doppler was placed between the tips of the mitral leaflets in the apical four-chamber view, and then diastolic functions of the left ventricle were measured (peak early [*E*] and late [*A*] diastolic wave velocities of the mitral valve as well as *E*/*A* ratio). All measurements were compared with the normal values of LVPW, IVS, LVES, and LVED which were taken from the standard tables according to ages and BSA [11]. An EF of less than 55% and FS of less than 29% were considered abnormal [12].

*(b) Tissue Doppler Imaging (TDI).* TDI was obtained according to the methods described by Kapusta et al. [13]. TDI studies were performed by the same echocardiography device. Apical four-chamber views were obtained and longitudinal peak annular velocity ratio was measured at lateral annulus of the mitral valve (pulsed tissue Doppler mode).

In the parasternal, long-axis view measurements of peak myocardial velocities were made guided by color coded TDI. Peak myocardial velocity during systole (*S*′), early diastole (*E*′), and late diastole (*A*′) was measured at the right and left ventricular free walls (RVFW and LVFW), respectively. Peak longitudinal myocardial velocities (*S*′, *E*′, and *A*′) were assessed within basal, middle, and apical parts of the right ventricular anterior wall and left ventricular posterior wall.

*(c) NT-proBNP Analysis*. Venous blood samples were obtained from an indwelling catheter after 30 minutes of rest in supine position. The blood samples were withdrawn into chilled tubes containing EDTA. The whole blood was centrifuged; plasma was decanted, immediately frozen, and stored at −27°C until assayed (within 6 months after sampling). Plasma concentration of NT-proBNP was measured by electrochemiluminescence immunoassay. It was performed with Modular *E*, using the NT-proBNP (Roche Diagnostics, Mannheim, Germany). The abnormal level was defined in our survivors based on age-dependent reference values by Albers and his colleagues [14].

### 2.1. Statistical Analysis

Data were analyzed using Microsoft Office 2007 (Excel) and Statistical Package for Social Science (SPSS) version 19.0.0, SPSS Inc., Chicago, IL, USA. Data were summarized using the arithmetic mean, standard deviation (SD), screening test, Studen's *t*-test, Chi-square test (*χ* ^2^), and Mann-Whitney *U* test. Probability (*P*) value was considered for statistical significance if it was less than 0.05.

## 3. Results

### 3.1. Subjects Characteristics

Our study included 80 children who were treated for childhood cancer and received anthracycline chemotherapeutic agents in their treatment protocols. None of the survivors had a history of acute cardiotoxicity following anthracycline dosage. Males and females were equally distributed, and the mean age at diagnosis was 5.62 + 1.72 years, while the mean duration of follow-up was 3.94 ± 1.37 years. The range of cumulative anthracycline doses was 175–380 mg/m^2^. Thirty-two patients (40%) had Hodgkin lymphoma, 27 patients (33.75%) had non-Hodgkin's lymphoma, and 21 patients (26.25%) had solid tumors (Wilms' tumor, neuroblastoma, and Rhabdomyosarcoma) (Table 1).

### 3.2. NT-proBNP Level

**Figure 1:**
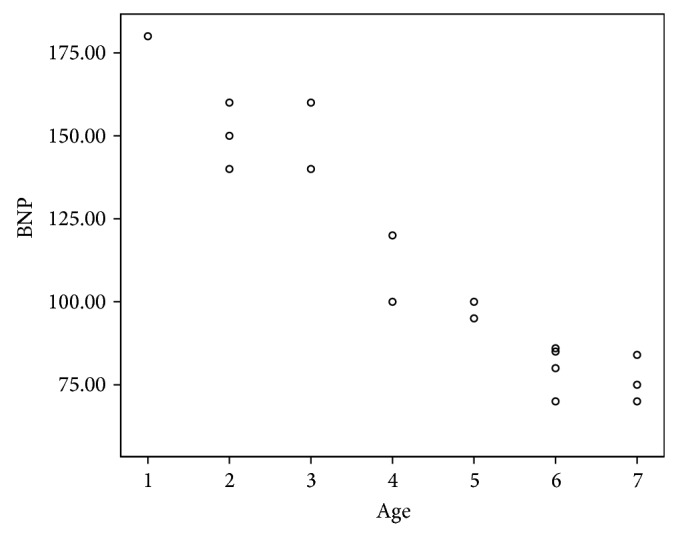
The correlation between NT-proBNP and age of patients.

**Figure 2:**
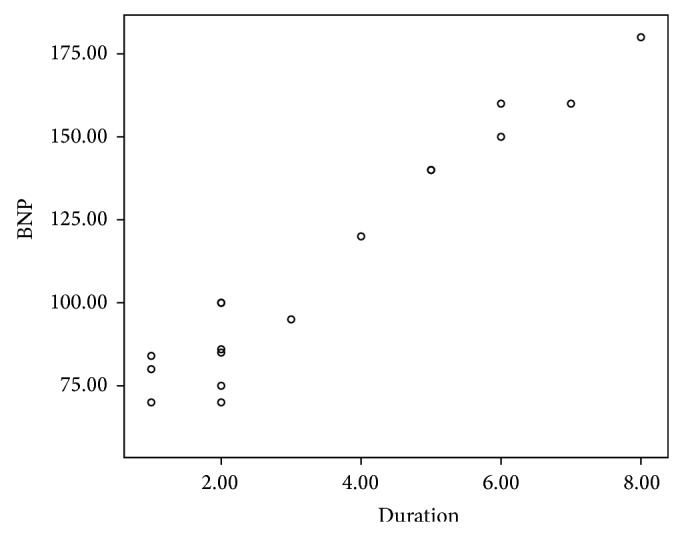
The correlation between NT-proBNP and duration of follow-up.

**Figure 3:**
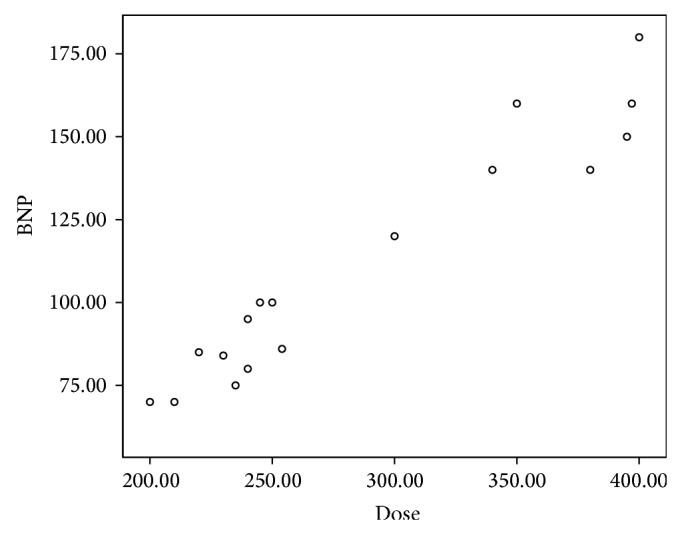
The correlation between NT-proBNP and cumulative dose of anthracycline.

In the present study, 24 patients representing 30% of the studied children showed abnormally high levels of NT-proBNP. There was no significant relation between abnormal NT-proBNP levels and the gender of patients. However, abnormal NT-proBNP levels were significantly related to age at diagnosis, duration of follow-up, and cumulative anthracycline dosage (*P* < 0.001). Abnormal NT-proBNP levels were associated with younger age of patients, longer duration of follow-up, and higher cumulative anthracycline doses (Figures 1, 2, and 3).

### 3.3. Echocardiographic Parameters and NT-proBNP

Echocardiographic parameters were obtained from all 80 survivors. Twelve patients (15%) had abnormal echocardiography. Abnormal left ventricular systolic function (EF and FS) and diastolic function (*E* velocity and *E*/*A* ratio) were not significantly related to abnormal NT-proBNP levels. *E* velocity was 115 ± 15 cm/s in abnormal NT-proBNP group versus 110 ± 13 cm/s in normal NT-proBNP group (*P* value > 0.05). *E*/*A* ratio was 1.91 ± 0.31 in abnormal proBNP group versus 1.83 ± 0.21 in normal proBNP group (*P* value > 0.05).

### 3.4. Tissue Doppler Imaging and NT-proBNP

Abnormal tissue Doppler imaging was diagnosed in 28 (35%) of the studied children.

**Figure 4:**
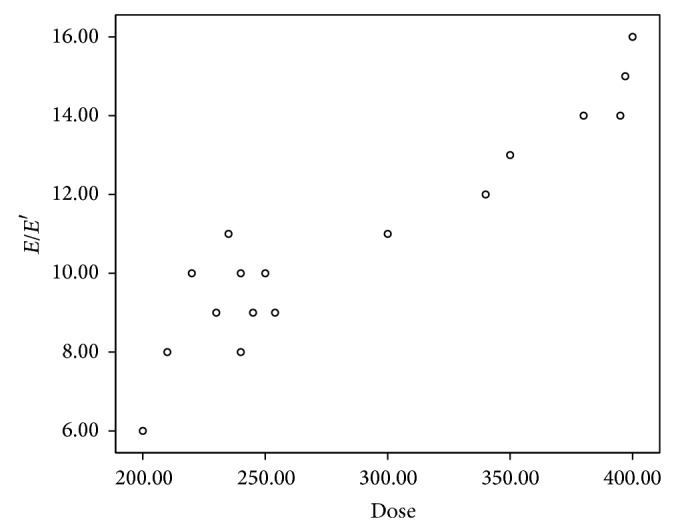
The correlation between diastolic function by TDI functions (*E*/*E*′) and cumulative dose of anthracycline.

Abnormal diastolic functions by TDI (*E*/*E*′) were positively correlated with cumulative dose of anthracyclines (Figure 4) and duration of follow-up and negatively correlated with age of patients.

**Figure 5:**
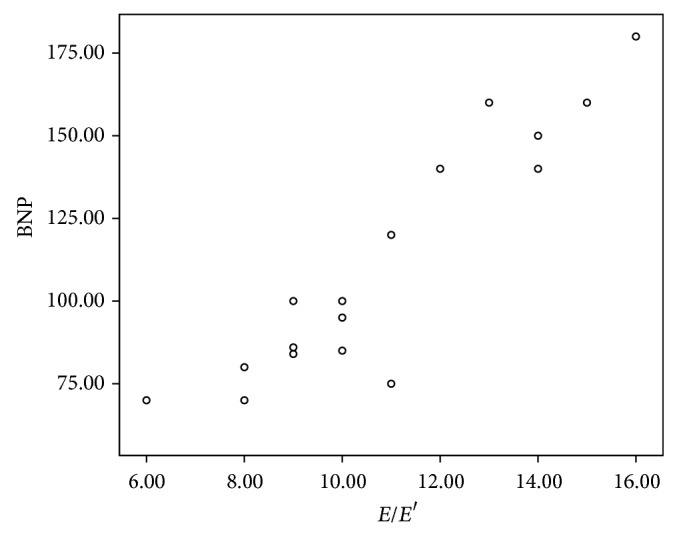
The correlation between proBNP and diastolic functions (*E*/*E*′).

There was a significant difference in LV diastolic function by pulsed tissue Doppler at lateral mitral annulus between normal and abnormal NT-proBNP groups. *E*′ (cm/s) was 8.18 ± 0.7 15 in abnormal NT-proBNP group versus 8.6 ± 0.6 in normal NT-proBNP group (*P* value 0.007) and *E*/*E*′ was 14.06 ± 0.97 in abnormal NT-proBNP group versus 12.79 ± 1.03 in normal NT-proBNP group (*P* value < 0.0001) (Figure 5).

**Figure 6:**
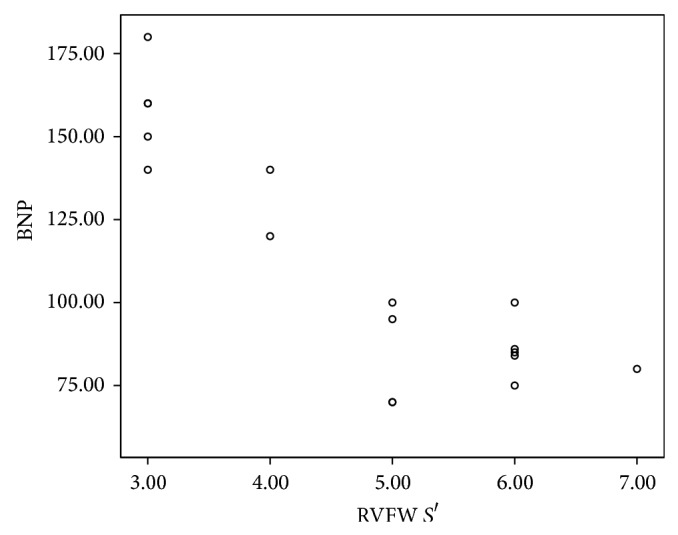
The correlation between NT-proBNP and RV systolic functions by TDI functions (*S*′).

**Figure 7:**
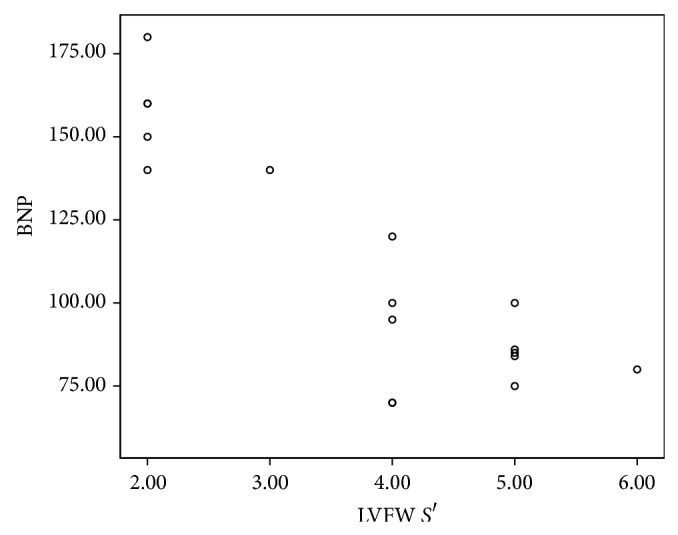
The correlation between NT-proBNP and LV systolic functions by TDI functions (*S*′).

There was a significant relation between abnormal NT-proBNP and peak myocardial velocities of right ventricle (RV) free wall using color coded TDI. Patients with increased level of NT-proBNP had significantly low systolic (*S*′) (Figure 6) and diastolic (*E*′ and *A*′) velocities in all right ventricular free wall segments with *P* value < 0.001 in most of them. There was a significant relation between abnormal NT-proBNP and myocardial velocities of LV posterior wall. *S*′ (Figure 7) and *A*′ velocities of the LV posterior wall were significantly lower in apical, middle, and basal parts. *E*′ velocity was significantly low in mid segment of LV posterior wall in abnormal proBNP group versus normal proBNP group (*P* value < 0.001); meanwhile there was no significant difference in *E*′ velocity of apical and basal parts of LV posterior wall between both groups (Table 2).

**Figure 8:**
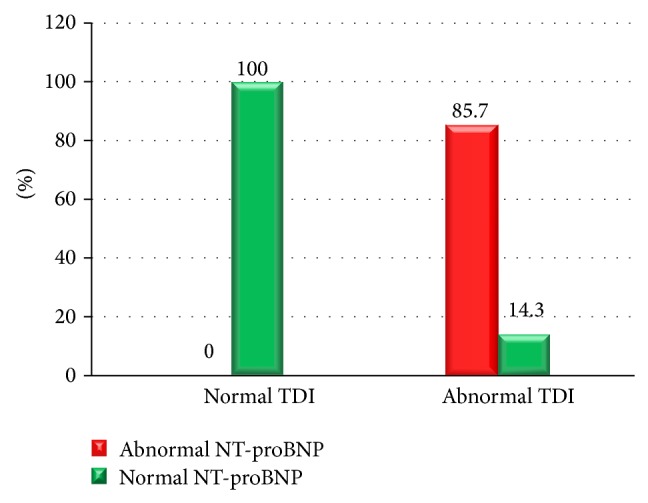
Relation between TDI and group with abnormal NT-proBNP and those with normal NT-proBNP.

Our data showed a significant relationship between NT-proBNP and TDI parameters as all patients with increased serum level of NT-proBNP showed abnormalities in TDI, while only 4 out of 56 patients with normal level of NT-proBNP had abnormal TDI findings. None of patients with normal TDI showed high levels of NT-proBNP (Figure 8).

Furthermore, our patients were classified into an unexposed group (48 patients who received only chemotherapy) and exposed group (32 patients who received both chemotherapy and radiotherapy). Our data showed that the level of NT-proBNP was significantly higher in the exposed group when compared with the unexposed group. NT-proBNP level was 144.3 ± 28.6 pg/mL in exposed group versus 115 ± 13.7 pg/mL in unexposed group (*P* value < 0.001). Regarding diastolic function assessment in study groups, there was no significant difference in early diastolic velocity by routine echocardiography between exposed and unexposed groups. *E* velocity (cm/s) was 82 ± 10 versus 79 ± 11 in unexposed group (*P* value 0.219); however there was a highly significant difference between both groups regarding diastolic function by TDI. *E*′ velocity (cm/s) was 5.7 ± 0. 8 in exposed group versus 8 ± 1.1 in unexposed group (*P* value < 0.0001) and *E*/*E*′ ratio was 14.38 ± 0.92 in exposed group versus 9.87 ± 1.32 in unexposed group (*P* value 0.0001) (Table 3). Compared to the unexposed group, all peak myocardial velocities (*S*′, *E*′, and *A*′) of different segments of LV posterior wall and RV anterior wall using color coded TDI were significantly lower in the exposed group (Table 4).

## 4. Discussion

Anthracycline chemotherapy and mediastinal and neck radiation are the most common causes of therapy-related cardiovascular complications in childhood cancer survivors [15]. The early identification of patients at risk for cardiotoxicity is a primary goal for both cardiologists and oncologists, allowing for the planning of personalized antineoplastic therapeutic strategies, the support of cardiac function, and the monitoring of the progression of cardiac damage [16]. There is a growing interest in the use of biomarkers for detection of anthracycline-induced cardiotoxicity. Natriuretic peptides are released by the myocardium in response to volume and pressure overload [17]. Although the role of NT-proBNP in the early detection of myocardial damage after anticancer therapy has been evaluated in several studies, the focus was mainly on level of this biomarker during or several months after therapy (7 and 18–22). In this study, NT-proBNP was used as a noninvasive technique for detection of late subclinical cardiotoxicity in survivors of childhood cancer. Abnormal NT-proBNP levels were detected in 30% of our asymptomatic survivors. This is in agreement with Mavinkurve-Groothuis et al. who found abnormal levels of NT-proBNP in 13% of 122 asymptomatic survivors of childhood cancer [18]. Moreover, the study of Sherief et al. detected an abnormal level of NT-proBNP in 20% of 50 of asymptomatic survivors [19]. Higher incidence in the current study may be related to the combined effects of anthracycline and radiotherapy as 40% of patients were exposed to both chemotherapy and radiotherapy. This result was in accordance with Adams and Lipshultz who demonstrated that concomitant cardiac irradiation has been recognized as risk factor of anthracycline cardiotoxicity [20]. Radiation may worsen the cardiotoxic effects of anthracycline, but whether this effect is additive or synergistic is unclear. Moreover, in the current study, the group exposed to both chemotherapy and radiotherapy showed higher levels of NT-proBNP than the unexposed group which was not exposed to radiotherapy. This is in agreement with D'Errico et al. who demonstrated increased level of NT-proBNP after radiotherapy when compared with nonirradiated patients [21]. Adams and Lipshultz demonstrated that concomitant cardiac irradiation has been recognized as a risk factor of anthracyclines cardiotoxicity [20]. This effect was explained by Braunwald who demonstrated that proinflammatory cytokines such as TNF and IL-6 were expressed in Reed-Sternberg cells from patients with Hodgkin disease (patients received chemotherapy and radiotherapy). These proinflammatory cytokines stimulate synthesis and secretion of NT-proBNP; at the same time they have been reported to impair cardiac function, several pathways are involved, including induction of apoptosis of cardiac myocytes by TNF type 1 and FAS activation [22]. Analysis of NT-proBNP in relation to risk factors of cardiotoxicity showed that abnormalities in NT-proBNP were significantly related to age at diagnosis, duration of follow-up, and cumulative anthracycline doses. The patients who had abnormal NT-proBNP were younger at diagnosis, had longer duration of follow-up, and received higher doses of anthracycline. The higher risks in the patients treated at younger age may be explained by immature cardiovascular tissues which are more vulnerable to chemotherapy and radiotherapy [23, 24]. These results were consistent with other authors [19, 25, 26]. On the contrary, Mavinkurve-Groothuis et al. found no significant relation between abnormal NT-proBNP levels and age at diagnosis and follow-up duration, but abnormal NT-proBNP levels were significantly related to cumulative anthracycline dosage [18]. Regarding the sex, our study showed no statistically significant difference regarding sex and NT-proBNP level. This result is in agreement with Mavinkurve-Groothuis et al. [18] but in disagreement with that reported by Lipshultz et al. who detected sex-related differences in NT-proBNP levels and reported that female survivors are more vulnerable to anticancer cardiotoxicity [27]. Moreover, Bu'Lock et al. reported that girls are significantly at greater risk than boys for late depressed contractility even when receiving the same cumulative dose of doxorubicin [28]. The importance of continuing to follow children with, or at risk for, premature symptomatic cardiovascular disease cannot be overemphasized. With longer follow-up after anthracycline treatment, the prevalence and severity of cardiac abnormalities increase [29–31]. Similar results were reported in the current work. Thus, the importance of lifetime follow-up cannot be overstated and preventing late cardiotoxicity must be a research priority [31, 32], particularly as the number of asymptomatic cancer survivors at risk for cardiac dysfunction later in life increases. More than 6 years of follow-up is necessary to identify those long term survivors at risk for cardiac dysfunction [31].

One of the main risk factors for anthracycline cardiotoxicity is high cumulative dose which is associated with a higher incidence of subclinical cardiac dysfunction [33]. In the current study, an abnormal level of NT-proBNP was significantly related to increasing cumulative anthracycline dose. According to our information, other studies reported that abnormal levels of NT-proBNP were significantly related to cumulative anthracycline dose [6, 18, 34]. On the other hand, Barry et al. and Gianni et al. reported that no dose of anthracyclines is free of cardiotoxicity with increasing duration of follow-up [35, 36].

NT-proBNP has been shown to be a sensitive marker for heart failure in children in earlier studies [37–39]. In patients with cardiac dysfunction, NT-proBNP may be used for diagnosis, treatment monitoring, and prognosis implications [39, 40]. There have been few studies concerning the relationship between the serum NT-proBNP level and echocardiographic parameters. In the study conducted by Germanakis et al. higher NT-proBNP levels were associated with reduced LV mass in asymptomatic children treated with anthracyclines [41]. In other studies, abnormal NT-proBNP levels were found to be significantly related to the end-diastolic LV internal diameter [18, 41]. In their studies, Hongkan et al. and Pongprot et al. found a significant relation between the NT-proBNP and diastolic parameters [42, 43]. Meanwhile, Brouwer et al. reported that abnormal FS and/or abnormal diastolic functions were present in 43% of adult childhood cancer survivors. Their NT-proBNP levels were higher in association with increased wall motion score index [44]. Also Dorup et al. found a lower *E* velocity in childhood cancer survivors exposed to anthracyclines [45]. In contrast to these studies, our study did not reveal any significant difference in systolic or diastolic function at the level of routine echocardiography between normal and abnormal NT-proBNP groups. This result is supported by Urbanova et al. [10] who could not reveal any echocardiographic changes in anthracycline treated patients with high NT-proBNP levels. Such differences between several studies are acceptable because there are various factors affecting the degree of cardiotoxicity, such as cancer type, cumulative anthracycline doses exposed, the age of the patient at the time of diagnosis, the time since the last chemotherapy, additional cardiotoxic medication, and history of mediastinal radiotherapy. Therefore, depending on the degree of myocardial injury, different correlations may be found between NT-proBNP levels and echocardiographic parameters.

TDI has been shown to be sensitive to identify anthracycline-induced cardiomyopathy and aberration in TDI parameters may be identified before abnormalities can be detected by conventional echocardiography [46]. In our study, we found that 35% of our survivors had global myocardial damage in the form of significant aberrations in peak myocardial velocities. Abnormal peak myocardial velocities were found in all cardiac cycles (*S*′, *E*′, and *A*′) in both LV and RV in some of our survivors in spite of normal routine echocardiography. Thus, TDI aberration was detected in hearts that appeared normal by routine echocardiography and might precede structural changes [46, 47]. Similar results were reported by Rathe et al. [48]. Aberration in TDI parameters has been shown to be highly sensitive to identify anthracycline-induced cardiomyopathy [46]. Moreover, all peak myocardial velocities (*S*′, *E*′, and *A*′ velocities) of different segments of LV posterior wall and RV free wall using color coded TDI were significantly lower in the group exposed to both anthracyclines and mediastinal irradiation when compared to the group exposed to anthracyclines only. This is in agreement with the study done by Adams and Lipshultz who demonstrated that concomitant cardiac irradiation has been recognized as a risk factor of anthracyclines cardiotoxicity [20].

Analysis of TDI parameters in relation to NT-proBNP showed a significant relation between elevated NT-proBNP levels and TDI abnormalities. Similar results were reported by Sherief et al. and Yildirim et al. [19, 49]. Tissue Doppler early diastolic velocity of the mitral annulus (*E*′) reflects the rate of myocardial relaxation, and it has been postulated as a good indicator of LV myocardial diastolic function. Normally, *E*/*E*′ at rest and during exercise (*E*/*E*′ < 8) are similar. Decreased mitral annular *E*′ is one of the earliest markers of diastolic dysfunction. In early diastolic dysfunction, TDI mitral annular early diastolic function (*E*′) is disturbed, whereas *E* velocity remains normal [50, 51]. In our study we found a significantly low *E*′ velocity in lateral mitral annulus with high *E*/*E*′ ratio in all cancer survivors with significantly higher *E*/*E*′ ratio in abnormal NT-proBNP group and the group exposed to combined chemotherapy and radiotherapy which suggest the role of TDI and NT-proBNP in early detection of late cardiotoxicity in childhood cancer survivors exposed to anthracyclines and in particular when combined with mediastinal irradiation. To the best of our knowledge, the only paper that studied the role of *E*/*E*′ ratio for detection of diastolic dysfunction in childhood cancer survivors exposed to anthracyclines was that done by Yildirim et al. who found a significant reduction in mitral septal annular early diastolic velocities *E*′ and elevation of mitral septal *E*/*E*′ values in a group exposed to anthracyclines [49]. However, Yildirim et al. rolled out patients exposed to mediastinal irradiation from the study [49], but we included them as a separate group, so, to the best of our knowledge, our study is the first to investigate the role of NT-proBNP and TDI (both pulsed and color coded) for detection of late cardiotoxicity induced by anthracyclines and mediastinal irradiation in childhood cancer survivors. This suggests the clinical usefulness of proBNP and TDI in detection of anthracycline and radiotherapy-induced cardiotoxicity and highlights their role as early markers of cardiotoxicity induced by anthracycline and mediastinal irradiation. Although these methods are promising, they have not been validated as surrogate end points for clinical toxicity and they are not routinely performed. We recommend prospective and multicenter studies, including large populations, using well-standardized methods for NT-proBNP and well-defined timing of sampling and cardiologic end point to validate the role of NT-proBNP as an early marker for late onset doxorubicin-induced cardiotoxicity.

## Figures and Tables

**Table 1:** Characteristics of 80 asymptomatic survivors of childhood cancer.

Total number of study population	**80**
Gender	
Male	40
Female	40
Age at diagnosis (years)	
Mean ± SD (range)	5.62 ± 1.72 (3–11)
Duration of follow-up (years)	
Mean ± SD (range)	3.94 ± 1.37 (2–7)
CAD (mg/m^2^)	
Mean ± SD (range)	245.4 ± 57.7 (175–380)
Diagnosis *n* (%)	
Hodgkin's lymphoma	32 (40)
Non-Hodgkin's lymphoma	27 (33.75)
Solid tumors	21 (26.25)
Wilms' tumor	11 (52.4)
Neuroblastoma	7 (33.3)
Rhabdomyosarcoma	3 (14.3)

CAD: cumulative anthracycline doses.

**Table 2:** Right and left ventricular TDI parameters in groups with normal and abnormal NT-proBNP.

	Systole (*S*′)			Diastole (*E*′)			Diastole (*A*′)		
	Abnormal *N* = 24	Normal *N* = 56	*P*	Abnormal *N* = 24	Normal *N* = 56	*P*	Abnormal *N* = 24	Normal *N* = 56	*P*
RVFW a	3.99 ± 0.81	5.03 ± 0.19	<0.001	5.35 ± 1.52	7 ± 0.68	<0.001	2.31 ± 0.7	3.22 ± 0.47	<0.001
RVFW m	6.03 ± 1.7	7.2 ± 0.47	<0.001	8.64 ± 1.88	10.4 ± 0.63	<0.001	4.35 ± 1.41	6.67 ± 0.52	<0.001
RVFW b	7.37 ± 1.62	8.75 ± 0.4	<0.001	10.79 ± 2.2	12.1 ± 0,86	<0.05	5.48 ± 1.1	7.14 ± 0.45	<0.001
LVFW a	3.28 ± 0.5	3.89 ± 0.27	<0.001	5.5 ± 0.95	5.7 ± 0.47	NS	2.31 ± 0.72	3.3 ± 0.32	<0.001
LVFW m	4.27 ± 0.75	5.85 ± +0.62	<0.001	7.6 ± 1.98	9.7 ± 0.58	<0.001	3.18 ± 0.55	4.2 ± 0.56	<0.001
LVFW b	5.6 ± 1.1	6.5 ± 1.03	<0.05	10.48 ± 2	10.42 ± 0.8	NS	4.72 ± 0.75	5.31 ± 0.79	<0.05

RVFW a, m, b: right ventricular free wall at the apical, middle, and basal parts, respectively.

LVFW a, m, b: left ventricular free wall at the apical, middle, and basal parts, respectively.

TDI: tissue Doppler imaging.

**Table 3:** Left ventricular diastolic function indices by echocardiography and pulsed TDI and NT-proBNP in exposed and unexposed study groups.

	Unexposed group	Exposed group	*P* value
NT-proBNP (pg/mL)	115 ± 13.7	144.3 ± 28.6	<0.001

Echo parameters			
*E*	79 ± 11	82 ± 10	0.219
*E*′	8 ± 0.11	5.7 ± 0.8	0.0001
*E*/*E*′	9.87 ± 1.32	14.38 ± 0.92	0.0001

Systole, *E*: early rapid filling during diastole, *E*′: early diastole, and *A*: late diastole.

**Table 4:** Right and left ventricular TDI parameters in exposed and unexposed study groups.

	Systole			Diastole (*E*)			Diastole (*A*)		
	Unexposed group	Exposed group	*P*	Unexposed group	Exposed group	*P*	Unexposed group	Exposed group	*P*
RVFW a	5.02 ± 0.2	4.2 ± 0.87	0.000	5.03 ± 0.2	4.3 ± 0.86	0.001	5.06 ± 0.4	4.3 ± 0.52	0.000
RVFW m	7.2 ± 0.51	6.4 ± 1.67	0.018	6.69 ± 0.54	5.02 ± 1.52	0.000	6.69 ± 0.54	5.02 ± 1.52	0.000
RVFW b	8.9 ± 0.25	7.71 ± 1.5	0.000	7.27 ± 0.43	6.8 ± 0.42	0.000	7.27 ± 0.34	5.9 ± 1.2	0.000
LVFW a	3.8 ± 0.29	3.4 ± 0.51	0.001	3.41 ± 0.32	2.59 ± 0.79	0.000	3.41 ± 0.32	2.59 ± 0.79	0.000
LVFW m	5.57 ± 0.36	4.58 ± 0.86	0.000	3.9 ± 0.29	3.4 ± 0.64	0.002	3.9 ± 0.37	3.4 ± 0.64	0.002
LVFW b	7.07 + 0.44	5.9 ± 1.12	0.000	5.7 ± 0.48	4.9 ± 0.95	0.001	5.07 ± 0.48	4.58 ± 0.86	0.001

RVW a, m, b: right ventricular free wall at the apical, middle, and basal parts, respectively.

LVW a, m, b: left ventricular free wall at the apical, middle, and basal parts, respectively.

TDI: tissue Doppler imaging.

Exposed: to both chemotherapy and radiotherapy.

Unexposed: only chemotherapy.
